# Altered Gene Expression Pattern in Peripheral Blood Mononuclear Cells in Patients with Acute Myocardial Infarction

**DOI:** 10.1371/journal.pone.0050054

**Published:** 2012-11-21

**Authors:** Marek Kiliszek, Beata Burzynska, Marcin Michalak, Monika Gora, Aleksandra Winkler, Agata Maciejak, Agata Leszczynska, Ewa Gajda, Janusz Kochanowski, Grzegorz Opolski

**Affiliations:** 1 First Chair and Department of Cardiology, Medical University of Warsaw, Warsaw, Poland; 2 Institute of Biochemistry and Biophysics, Polish Academy of Sciences, Warsaw, Poland; Virginia Commonwealth University Medical Center, United States of America

## Abstract

**Background:**

Despite a substantial progress in diagnosis and therapy, acute myocardial infarction (MI) is a major cause of mortality in the general population. A novel insight into the pathophysiology of myocardial infarction obtained by studying gene expression should help to discover novel biomarkers of MI and to suggest novel strategies of therapy. The aim of our study was to establish gene expression patterns in leukocytes from acute myocardial infarction patients.

**Methods and Results:**

Twenty-eight patients with ST-segment elevation myocardial infarction (STEMI) were included. The blood was collected on the 1^st^ day of myocardial infarction, after 4–6 days, and after 6 months. Control group comprised 14 patients with stable coronary artery disease, without history of myocardial infarction. Gene expression analysis was performed with Affymetrix Human Gene 1.0 ST microarrays and GCS3000 TG system. Lists of genes showing altered expression levels (fold change >1.5, p<0.05) were submitted to Ingenuity Pathway Analysis. Gene lists from each group were examined for canonical pathways and molecular and cellular functions. Comparing acute phase of MI with the same patients after 6 months (stable phase) and with control group we found 24 genes with changed expression. In canonical analysis three pathways were highlighted: signaling of PPAR (peroxisome proliferator-activated receptor), IL-10 and IL-6 (interleukin 10 and 6).

**Conclusions:**

In the acute phase of STEMI, dozens of genes from several pathways linked with lipid/glucose metabolism, platelet function and atherosclerotic plaque stability show altered expression. Up-regulation of SOCS3 and FAM20 genes in the first days of myocardial infarction is observed in the vast majority of patients.

## Introduction

Acute myocardial infarction (MI) remains the leading cause of death despite the substantial progress in diagnosis and therapy in recent decades. In the acute phase of MI increased leukocyte count, a non-specific marker of inflammation, is the risk factor for future cardiovascular events and predicts mortality in those with STEMI [ST-segment elevation MI], NSTEMI (non-STEMI) or unstable angina [1], [2]. It has also been shown that an elevated leukocyte count predicts 1-year mortality independently of the risk factors for coronary artery disease across the entire spectrum of acute coronary syndromes (ACS) [3]. The mechanisms linking activation of inflammation and ACS are complex – inflammation seems to be linked to the initiation and progression of atherosclerosis [4]. Obtaining novel insights into the pathophysiology of myocardial infarction by analyzing gene expression patterns in leucocytes should aid the discovery of novel biomarkers of MI and elaboration of novel therapeutic strategies. The aim of our pilot study was the first attempt at establishing leukocyte gene expression signatures of the acute phase of MI.

## Materials and Methods

### Patients

Patients presenting with STEMI were included in the Ist Chair and Departament of Cardiology of Medical University of Warsaw in 2010. We sought to include consecutive patients that agreed to participate in the study (due to technical aspects of blood collection, only patients admitted between Sunday and Thursday were taken into consideration). All the patients underwent coronary angiography and angioplasty of infarct related artery. Pharmacological treatment was according to current guidelines [5]. Blood was collected on the 1^st^ day of myocardial infarction (admission), after 4–6 days (discharge), and after 6 months. Participation in the study had no influence on the pharmacological treatment and procedures underwent by the patients. Control group comprised patients with proven coronary artery disease: with coronary angiography (at least one stenosis exceeding 50% or previous coronary angioplasty of previous coronary artery bypass graft), or with non-invasive tests (positive exercise test) and no history of myocardial infarction. The study was approved by the Bioethics Committee of the Medical University of Warsaw and all patients gave written informed consent.

**Table 1:** Major characteristics of the study and control groups.

	Study group	Control group	p-value
Men/Women	21/7	11/3	0.89
Age*	55.4±10.2	67.8±7.4	0.0003
Anterior myocardialinfarction**	14 (50%)	NA	NA
Hypertension**	13 (46.4%)	10 (71.4%)	0.23
Diabetes**	5 (17.9%)	3 (21.4%)	0.89
Current smoker**	15 (53.6%)	4 (28.6%)	0.23
Previous myocardialinfarction**	1 (3.6%)	NA	NA
Body Mass Index (BMI)*	29.4±4.4	29.5±4.8	0.58
Left ventricular EF (%)*	46.7±8.7	59.0±6.0	0.011
Treatment			
Aspirin**	28 (100%)	12 (85.7%)	0.20
Clopidogrel**	28 (100%)	3 (21.4%)	<0.0001
Statin**	28 (100%)	13 (92.8)	0.72
Beta blocker**	28 (100%)	13 (92.8)	0.72
ACE inhibitor**	20 (71.4%)	12 (85.7%)	0.52
Heparin (UFH orLMWH)**	28 (100%)	0 (0%)	<0.0001

* Mean ± Standard Deviation.

** number (%);

NA – not applicable.

### RNA Isolation

Sodium-heparinized blood was collected from 28 patients at the three time points. Peripheral blood mononuclear cells (PBMC) were purified using BD Vacutainer® CPT™ Cell Preparation Tube according to the manufacturer’s instructions (Becton, Dickinson and Co. Franklin Lakes, NJ,USA).

Total RNA was isolated from PBMC with the MagNA Pure Compact System (Roche Diagnostics GmbH, Germany) according to the manufacturer’s recommendations. RNA samples were quantified by UV absorption (Nanodrop, LabTech International, UK) and their quality was checked with the RNA 600 Nano Assay Kit using Bioanalyzer© in accordance with the manufacturer’s procedures (Agilent, Santa Clara, CA, USA). Samples with an RNA integrity number of eight or above were considered suitable for use in microarrays. RNA samples were stored at −80°C until further analysis.

**Table 2:** Differentially expressed genes common to both analyses: admission versus 6 months after MI and admission versus control.

Admission vs 6 months		Admission vs control			
Fold Change	p-value	Fold Change	p-value	Symbol	Entrez Gene Name
−1.505	1.36E−09	−1.664	7.16E–07	MYBL1	v-myb myeloblastosis viral oncogene homolog (avian)-like 1
−1.563	1.51E−07	−2.006	2.14E–05	KLRC4	killer cell lectin-like receptor subfamily C, member 4
−1.578	2.30E−06	−2.213	6.33E–05	KLRC2	killer cell lectin-like receptor subfamily C, member 2
1.518	5.04E−13	2.236	1.07E–02	TMEM176A	transmembrane protein 176A
1.570	1.68E−16	1.753	2.51E–08	ST14, MT-SP1/matriptase	suppression of tumorigenicity 14 (colon carcinoma)
1.573	5.36E−05	1.732	1.74E–04	HBEGF	heparin-binding EGF-like growth factor
1.577	5.82E−16	1.950	1.62E–09	ASGR2	asialoglycoprotein receptor 2
1.584	5.95E−14	1.989	3.39E–10	STAB1	stabilin 1
1.587	3.26E−03	1.605	2.50E–02	EGR2	early growth response 2
1.589	1.07E−06	1.555	1.48E–03	FMN1 (includes EG:296512)	formin 1
1.601	6.36E−12	1.533	2.03E–04	CR1	complement component (3b/4b) receptor 1 (Knops blood group)
1.611	3.11E−13	1.674	2.68E–05	RNASE1	ribonuclease, RNase A family, 1 (pancreatic)
1.629	1.88E−12	1.837	1.40E–06	TCN2	transcobalamin II
1.638	4.61E−11	1.512	1.62E–04	VSIG4	V-set and immunoglobulin domain containing 4
1.669	6.74E−12	1.586	3.42E–05	CYP1B1	cytochrome P450, family 1, subfamily B, polypeptide 1
1.693	6.24E−13	1.771	3.90E–05	DYSF	dysferlin, limb girdle muscular dystrophy 2B (autosomal recessive)
1.761	1.99E−11	1.769	1.33E–04	RNASE2	ribonuclease, RNase A family, 2 (liver, eosinophil-derived neurotoxin)
1.778	9.45E−13	1.711	1.84E–07	PPARG	peroxisome proliferator-activated receptor gamma
1.904	9.39E−11	1.631	1.25E–03	FCGR1A	Fc fragment of IgG, high affinity Ia, receptor (CD64)
1.945	1.01E−10	1.972	2.16E–05	FAM20A	family with sequence similarity 20, member A
1.958	1.71E−15	1.732	4.98E–05	AQP9	aquaporin 9
2.066	5.51E−05	1.955	4.17E–03	EGR1	early growth response 1
2.339	9.00E−14	2.008	4.14E–04	HP	haptoglobin
2.929	1.60E−20	2.443	5.10E–09	SOCS3	suppressor of cytokine signaling 3

**Figure 1:**
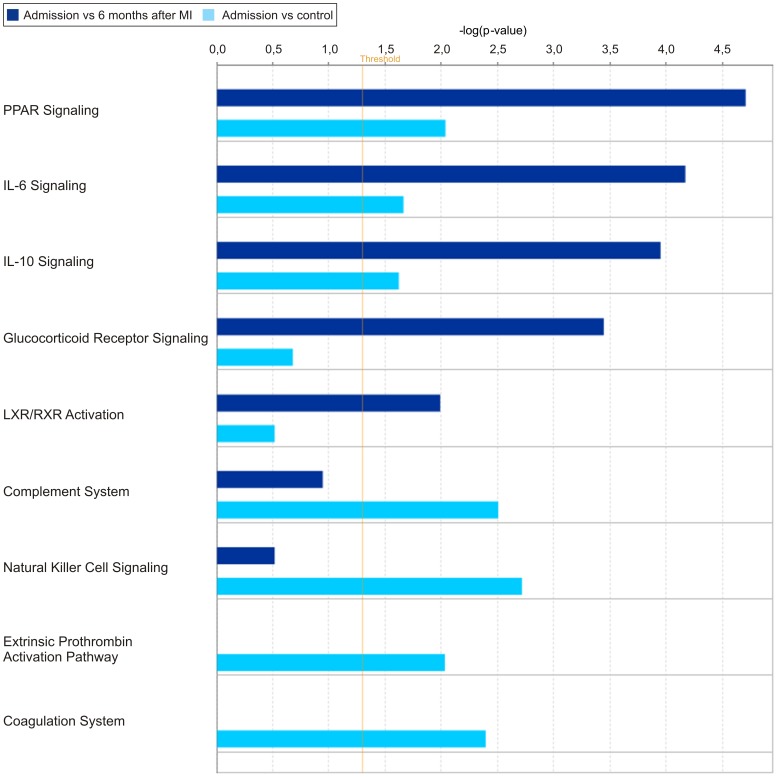
Top canonical pathways associated with acute phase of STEMI. Ingenuity Pathway Analysis of gene sets differentially expressed on the first day of myocardial infarction versus 6 months after MI or versus control group was performed. Functional categories are represented on the x-axis. The significance is expressed as the negative exponent on the p-value calculated for each function on the y-axis of the diagram, increasing with bar height.

**Table 3:** Validation of microarray data using qRT-PCR.

	Mean expression ratio (S.D.)	
Gene Symbol	Admission vs 6 months	Admission vs control
KLRC2	0.59 (0.43)^NS^	0.22 (0.13)^*^
MYBL1	0.55 (0.19)^NS^	0.56 (0.20)^*^
KLRC4	0.51 (0.34)^NS^	0.65 (0.41)^NS^
SOCS3	12.63 (5.23)***	5.42 (2.21)^**^
FAM20A	6.31 (3.09)***	3.56 (1.62)^*^
EGR1	5.82 (4.54)^*^	6.64 (5.85)^*^
PPARG	4.20 (1.71)***	4.28 (1.76)^**^
HP	4.00 (2.75)^NS^	3.12 (1.88)^NS^
HBEGF	3.79 (2.05)^*^	2.77 (1.53)^NS^
EGR2	3.68 (3.26)^NS^	6.93 (6.60)^NS^
VSIG4	3.62 (1.42)^**^	3.72 (1.53)^*^
AQP9	3.28 (1.34)^**^	2.44 (0.87)^*^
STAB1	2.97 (1.08)^*^	1.64 (0.45)^NS^
ST14	2.79 (0.81)***	1.61 (0.45)^ NS^
RNASE2	2.79 (1.06)^**^	2.08 (0.76)^NS^
CR1	2.67 (0.83)^**^	2.88 (1.01)^NS^
RNASE1	2.67 (0.91)^*^	3.07 (1.16)^NS^
CYP1B1	2.65 (0.77)^**^	1.78 (0.57)^NS^
FCGR1A	2.65 (1.07)^*^	1.92 (0.68)^NS^
ASGR2	2.49 (0.92)^*^	2.45 (0.83)^*^
DYSF	2.48 (1.05)^NS^	1.60 (0.58)^NS^
TCN2	2.05 (0.57)^*^	1.57 (0.42)^NS^
TMEM176A	2.00 (0.98)^NS^	8.77 (5.20)^**^
FMN1	1.72 (0.63)^NS^	3.64 (1.82)^NS^

*** p<0.001; **p<0.01; *p<0.05; ^NS^ – not significant.

All genes abbreviations are explained in Table 2.

**Table 4:** Differentially expressed genes common to both analyses: admission versus control and discharge versus control.

Admission vs control		Discharge vs control			
Fold Change	p-value	Fold Change	p-value	Symbol	Entrez Gene Name
−3.501	4.71E–19	−3.030	2.49E–16	SNHG12	small nucleolar RNA host gene 12 (non-protein coding)
−3.155	8.66E–23	−3.084	2.87E–22	SNORA24	small nucleolar RNA, H/ACA box 24
−3.090	3.79E–06	−3.118	3.24E–06	GPR128	G protein-coupled receptor 128
−2.777	1.40E–15	−2.479	2.52E–13	SNORA20	small nucleolar RNA, H/ACA box 20
−2.617	3.72E–05	−1.739	1.44E–02	CLC	Charcot-Leyden crystal protein
−2.448	4.05E–23	−12.134	5.82E–19	TAF1D	TATA box binding protein (TBP)-associated factor, RNA polymerase I, D, 41kDa
−2.308	1.07E–11	−2.086	8.97E–10	SNORD105	small nucleolar RNA, C/D box 105
−2.301	5.66E–12	−2.065	7.27E–10	ANKRD32	ankyrin repeat domain 32
−2.293	2.24E–16	−2.026	3.11E–13	SCARNA6	small Cajal body-specific RNA 6
−2.256	6.24E–15	−2.095	3.85E–13	RPL13A	ribosomal protein L13a
−2.227	1.22E–10	−2.062	3.14E–09	RABGGTB	Rab geranylgeranyltransferase, beta subunit
−2.213	6.33E–05	−1.938	7.42E–04	KLRC2	killer cell lectin-like receptor subfamily C, member 2
−2.110	4.52E–06	−1.615	2.36E–03	MS4A3	membrane-spanning 4-domains, subfamily A, member 3 (hematopoietic cell-specific)
−2.108	1.48E–07	−2.005	7.56E–07	SNORD82	small nucleolar RNA, C/D box 82
−2.102	4.49E–09	−1.960	6.93E–08	SNORD60	small nucleolar RNA, C/D box 60
−2.081	3.30E–18	−1.964	2.12E–16	SNORD50A	small nucleolar RNA, C/D box 50A
−2.063	6.32E–08	−1.904	1.04E–06	SNORD61	small nucleolar RNA, C/D box 61
−2.057	5.83E–09	−1.966	3.53E–08	SNHG3	small nucleolar RNA host gene 3 (non-protein coding)
−2.053	8.31E–20	−2.034	1.67E–19	SNORD15B	small nucleolar RNA, C/D box 15B
−2.047	1.29E–10	−1.949	1.30E–09	SNORD41	small nucleolar RNA, C/D box 41
−2.044	3.75E–18	−1.863	3.49E–15	SCARNA7	small Cajal body-specific RNA 7
−2.012	5.22E–06	−1.934	1.55E–05	RNU4-2	RNA, U4 small nuclear 2
−2.006	2.14E–05	−1.708	8.59E–04	KLRC4	killer cell lectin-like receptor subfamily C, member 4
−1.994	6.60E–12	−1.906	7.73E–11	SCARNA10	small Cajal body-specific RNA 10
−1.983	1.77E−10	–1.833	8.01E–09	SNORD1C	small nucleolar RNA, C/D box 1C
−1.982	1.44E–04	−1.709	2.52E–03	KLRC3	killer cell lectin-like receptor subfamily C, member 3
−1.972	7.41E–15	−1.848	5.48E–13	SNORA68	small nucleolar RNA, H/ACA box 68
−1.946	2.00E–12	−1.833	6.50E–11	SNORD54	small nucleolar RNA, C/D box 54
−1.944	4.72E–15	−1.915	1.33E–14	LLPH	LLP homolog, long-term synaptic facilitation (Aplysia)
−1.824	2.81E–12	−1.721	1.16E–10	RMRP	RNA component of mitochondrial RNA processing endoribonuclease
−1.784	1.75E–10	−1.672	7.20E–09	SCARNA5	small Cajal body-specific RNA 5
−1.770	2.35E–10	−1.705	2.07E–09	SNHG10	small nucleolar RNA host gene 10 (non-protein coding)
−1.755	3.86E–10	−1.602	6.87E–08	SNORA3	small nucleolar RNA, H/ACA box 3
−1.747	6.99E–08	−1.613	2.46E–06	SNORA23	small nucleolar RNA, H/ACA box 23
−1.744	6.49E–09	−1.517	6.28E–06	CEP78	centrosomal protein 78kDa
−1.727	3.33E–05	−1.505	1.54E–03	KLRC1	killer cell lectin-like receptor subfamily C, member 1
−1.726	7.94E–09	−1.507	6.80E–06	SNORD28	small nucleolar RNA, C/D box 28
−1.725	1.43E–10	−1.647	2.52E–09	HIST1H4A (includes others)	histone cluster 1, H4a
−1.725	5.62E–05	−1.506	2.07E–03	SNORD104	small nucleolar RNA, C/D box 104
−1.718	5.48E–05	−1.521	1.47E–03	DTHD1	death domain containing 1
−1.717	5.51E–07	−1.544	3.82E–05	SNHG1	small nucleolar RNA host gene 1 (non-protein coding)
−1.716	1.83E–16	−1.674	1.67E–15	SNORA37	small nucleolar RNA, H/ACA box 37
−1.694	2.56E–07	−1.635	1.27E–06	SNORD58A	small nucleolar RNA, C/D box 58A
−1.693	6.32E–08	−1.677	1.01E–07	RNU5A-1	RNA, U5A small nuclear 1
−1.689	1.23E–10	−1.585	7.26E–09	SNORD46	small nucleolar RNA, C/D box 46
−1.670	5.57E–07	−1.713	1.80E–07	COX7B	cytochrome c oxidase subunit VIIb
−1.649	3.92E–07	−1.514	1.74E–05	PRSS23	protease, serine, 23
−1.643	8.00E–09	−1.656	4.98E–09	WDR74	WD repeat domain 74
−1.619	5.23E–11	−1.633	2.76E–11	SNORD59B	small nucleolar RNA, C/D box 59B
−1.616	6.35E–11	−1.546	1.55E–09	RPL7A	ribosomal protein L7a
−1.600	1.28E–06	−1.505	1.91E–05	SNORD14E	small nucleolar RNA, C/D box 14E
−1.599	3.43E–08	−1.525	4.88E–07	SNORD50B	small nucleolar RNA, C/D box 50B
−1.592	2.70E–06	−1.556	7.26E–06	SNORD30	small nucleolar RNA, C/D box 30
−1.590	3.50E–09	−1.526	4.58E–08	SNORD6	small nucleolar RNA, C/D box 6
−1.589	2.19E–11	−1.629	3.11E–12	SNORD63	small nucleolar RNA, C/D box 63
−1.585	6.35E–08	−1.584	6.67E–08	SCARNA1	small Cajal body-specific RNA 1
−1.575	3.97E–07	−1.520	2.37E–06	SNORA22	small nucleolar RNA, H/ACA box 22
−1.550	1.03E–10	−1.593	1.19E–11	SNORA19	small nucleolar RNA, H/ACA box 19
−1.532	8.76E–12	−1.681	2.33E–15	BRK1	BRICK1, SCAR/WAVE actin-nucleating complex subunit
−1.531	2.32E–08	−1.624	4.74E–10	RNU4ATAC	RNA, U4atac small nuclear (U12-dependent splicing)
1.528	7.46E–05	1.554	4.03E–05	C1QB	complement component 1, q subcomponent, B chain
1.546	1.92E–06	1.567	9.90E–07	C1QC	complement component 1, q subcomponent, C chain
1.568	2.10E–14	1.552	5.55E–14	GPSM3	G-protein signaling modulator 3
1.586	3.42E–05	1.568	3.32E–05	CYP27A1	cytochrome P450, family 27, subfamily A, polypeptide 1
1.597	4.30E–07	1.572	9.32E–07	LGALS9B	lectin, galactoside-binding, soluble, 9B
1.620	7.19E–06	1.567	2.59E–05	CD151	CD151 molecule (Raph blood group)
1.629	5.86E–11	1.586	4.11E–10	RAB1B	RAB1B, member RAS oncogene family
1.678	1.30E–26	1.545	6.59E–22	MLF2	myeloid leukemia factor 2
1.708	1.09E–14	1.517	2.01E–10	TTYH3	tweety homolog 3 (Drosophila)
1.740	2.15E–13	1.584	2.24E–10	WAS	Wiskott-Aldrich syndrome (eczema-thrombocytopenia)
1.747	8.66E–11	1.517	3.89E–07	CA5BP1	carbonic anhydrase VB pseudogene 1
1.765	9.63E–08	1.634	2.64E–06	LGALS9	lectin, galactoside-binding, soluble, 9
1.915	9.75E–03	1.875	1.23E–02	GSTM1	glutathione S-transferase mu 1
1.950	1.62E–09	1.542	3.58E–05	ASGR2	asialoglycoprotein receptor 2
1.972	2.16E–05	1.588	3.00E–03	FAM20A	family with sequence similarity 20, member A

### cDNA Microarrays

RNA (100 ng) was reverse transcribed, amplified, and labeled with biotin using the whole transcript sense target labeling kit and hybridized for 16 h at 45°C to Human Gene 1.0 ST arrays (Affymetrix, Santa Clara,CA, USA), according to the manufacturer’s instructions. Following hybridization, the probe arrays were washed and stained on a fluidics station and immediately scanned on an Affymetrix GCS 3000 GeneArray Scanner.

**Figure 2:**
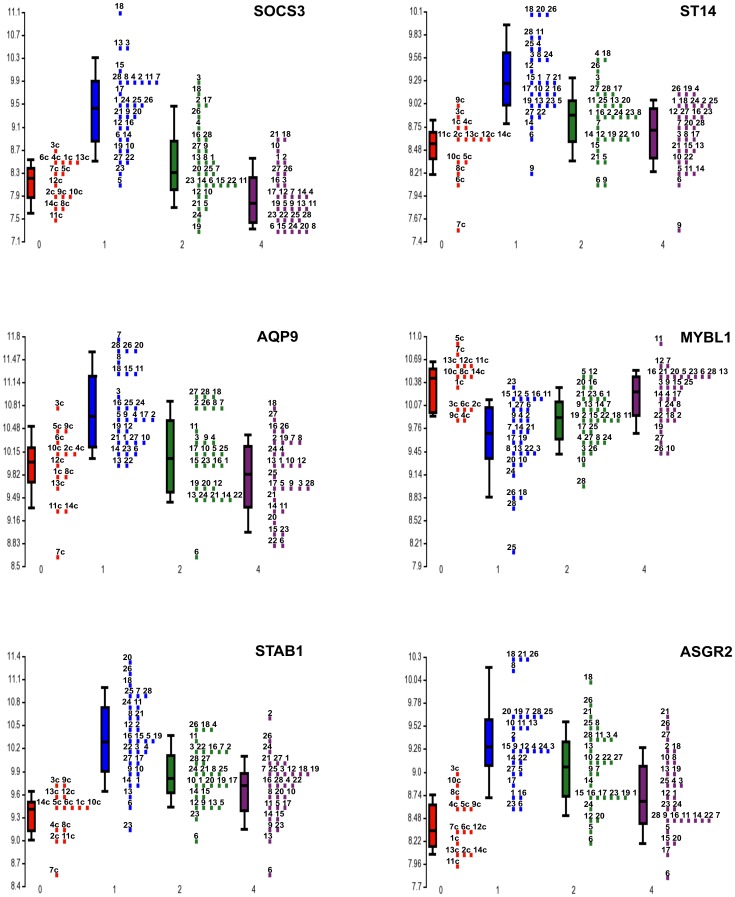
Expression data from microarray experiments for chosen genes. The y-axis represents the log2 normalized intensity of the gene and the x-axis represents analyzed groups. The line inside the box and whiskers represents the median of the samples in a group. Points present relative expression levels in individual patients at admission (blue), at discharge (green), 6 month after MI (violet) and from control group (red). Numbers indicate the coded identity of a particular patient. SOCS3– suppressor of cytokine signaling 3; ST14– MT-SP1/matriptase; AQP 9– aquaporin 9; MYBL1– v-myb myeloblastosis viral oncogene homolog (avian)-like 1; STAB1– stabilin 1; ASGR2– asialoglycoprotein receptor 2.

### Data Analysis of Microarrays

Quality controls were performed using Microarray Suite 5.0 software provided by Affymetrix (www.affymetrix.com) according to the manufacturer’s recommendations. Affymetrix raw gene array data were processed using the Partek Genomics Suite software (Partek Inc., St. Louis, MO, USA).

Comparisons were performed between MI group at day one of day 4–6 on the one hand and MI group at 6 months or control on the other. Lists of genes showing significant differences in expression levels between groups were submitted to Ingenuity Pathway Analysis (Ingenuity® Systems, www.ingenuity.com) for canonical pathways and subjected to network analyses.

### Determination of mRNA Levels by qRTPCR

Total RNA was converted into cDNA using the QuantiTect Reverse Transcription Kit (QIAGEN, Germany) according to the manufacturer’s recommendations.

qPCR amplification was performed using a using LightCycler 1.5 and LightCycler 480 Instruments, and LightCycler FastStart DNA Master SYBR Green I and LightCycler 480 SYBR Green I Master (Roche Diagnostics GmbH, Germany) according to the manufacturer’s instructions. The Pfaffl model [6] and the relative expression software tool (REST-MCS©-version 2) [7] were used to estimate changes in the relative mRNA levels of a 24 genes in order to validate the results obtained in the microarray study. We determined the relative expression levels of the selected genes at the 1^st^ day of myocardial infarction (on admission), 6 months after MI and in the control group, using samples from six randomly chosen patients from both groups. Data normalization was carried out against transcripts of the HPRT and TUBB genes. The sequences of all primers and qPCR amplification parameters are available in supplementary data (Table S1).

#### Statistical analysis

Continuous variables are presented as mean ±SD (standard deviation). Categorical variables are presented as frequencies. Statistical significance of the results was estimated by variance analysis (ANOVA). Differences were considered statistically significant at a nominal significance of p<0.05 and a fold change >1.5 in expression between the admission, discharge and control groups.

## Results

Twenty-eight patients with ST-segment elevation myocardial infarction (STEMI) were included. All the patients completed the follow-up visit 6 months after admission. The control group comprised 14 patients. Basic characteristics of the groups are shown in table 1.

### Admission

On the first day of MI 91 genes showed a significantly different level of expression compared with 6 months after MI. Of those, 57 were annotated, including three pairs of duplicate genes. Of the 54 unique annotated genes with altered expression 51 were up- and three down-regulated at day 1 versus 6 months (Table S2). In turn, a comparison of gene expression on the first day of MI with the control group identified 491 genes a significantly differentially expressed (212 annotated, including 14 pairs of duplicate genes, 85 were up- and 127 down-regulated, Table S3). Twenty-four genes were shared between the two comparisons (MI vs. after 6 months and MI vs. control, Table 2).

In a canonical analysis of the genes with altered expression several pathways were found, the most significant being: signaling of PPAR (peroxisomal proliferator-activated receptor), IL-6 (interleukin 6) signaling, and IL-10 (interleukin 10) signaling. These pathways were common for both comparisons (Figure 1).

Additionally, a comparison between day one and after 6 months revealed significant changes in glucocorticoid receptor signaling and activation of LXR/RXR (liver × receptor/retinoid × receptor). In contrast, analysis of the day one MI group versus the control highlited genes associated with Complement System, Coagulation System, Natural Killer Cell Signaling, and Extrinsic Prothrombin Activation Pathway.

In the molecular and cellular function categories Cellular Growth and Proliferation, Cell-To-Cell Signaling and Interaction, and Cell Movement were markedly changed in both analyses.

To validate the microarray results, mRNA levels of twenty-four genes shared between the two comparisons were quantified by qRT-PCR (Table 3). Results of validation confirmed the microarray data and for most of the genes the fold-changes calculated by qRT-PCR exceeded the microarray ones.

### Discharge

On the 4^th^–6^th^ day after MI expression of 34 genes was changed relative to 6 months after MI: 18 with annotation including two pairs and one gene represented by seven probes, which makes 10 unique genes, all upregulated (Table S4). In a canonical analysis five most significant pathways were: Primary Immunodeficiency Signaling, B Cell Development; Communication between Innate and Adaptive Immune Cells; Systemic Lupus Erythematosus Signaling; Role of Macrophages, Fibroblasts and Endothelial Cells in Rheumatoid Arthritis.

Analysis of 4^th^–6^th^ day after MI versus the control group indicated 302 differentially expressed genes with 98 of them annotated, including three pairs of duplicates; 26 up- and 72 down-regulated (Table S5). Association with Hematopoiesis from Pluripotent Stem Cells, Primary Immunodeficiency Signaling, Natural Killer Cell Signaling, Complement System, and EIF2 Signaling canonical pathways was found.

### Genes Shared between Admission and Discharge

Taking into account genes common to the two analyses of gene expression on admission and on discharge versus after 6 months, only two transcripts showed significantly altered expression: FAM20A and SOCS3. In contrast, when the same admission and discharge groups were compared with the control 75 genes were shared between the two comparisons, including 39 down-regulated small non-coding RNA transcripts (Table 4).

Additionally, we undertook a patient-by-patient analysis of the most differently expressed genes between all groups. The direction of change was the same in all patients although the relative levels of expression differed markedly between patients (Figure 2).

## Discussion

We have shown here that in leukocytes of STEMI patients tens of genes show altered expression during or soon after MI relative to the stable coronary artery disease. Interestingly, at the day of the infarct they include mainly genes and pathways directly or indirectly linked with lipid/glucose metabolism, platelet function and atherosclerotic plaque stability. On discharge, many genes related to specific immune response (encoding immunoglobulins) show altered expression. Comparing the admission and the discharge profiles with the control group, a strikingly high number of small non-coding RNA transcripts (most frequent small nucleolar RNA, snoRNA) show altered expression. Those snoRNAs are known to act as guide molecules for site-specific methylation and pseudouridylation of other RNAs. snoRNA-guided modifications of mRNA seem possible and could affect splicing, translation or mRNA stability. However, the clinical significance of snoRNA is not known [8].

### Gene Expression

Below we discuss selected literature references indicating a possible relevance of individual genes showing altered expression in this study (see Table 2) to the functioning of the cardiovascular system.

We have shown up-regulation of SOCS3 and FAM20 genes in the first 4–6 days of myocardial infarction. SOCS3 is one of the eight proteins of the SOCS group that block the JAK (Janus kinase) tyrosine kinase activity and the activation of STAT factors [9]. Expression of SOCS3 has been shown to be significantly reduced in balloon-injured porcine coronary arteries [10]. Recently it was reported that the progression of LV remodeling after AMI was prevented in SOCS3-deficient mice. SOCS3 deletion enhanced multiple cardioprotective signaling pathways including STAT3, AKT, and extracellular signal-regulated kinase (ERK)-1/2, while inhibiting myocardial apoptosis and fibrosis [11].

The FAM20 family comprises secreted proteins with potential roles in regulating differentiation and functioning of hematopoietic and other tissues. The FAM20A mRNA is only expressed during early stages of hematopoietic development [12]. The role of FAM198B is unknown.

ST14 (MT-SP1/matriptase) of plays roles in growth factor activation, receptor activation and inactivation, protease activation, and ectodomain shedding [13]. In atherosclerotic lesions, enhanced mRNA and protein expression of ST14 was found relative to nondiseased vessels [14]. Asialoglycoprotein receptor (ASGPR) is a hepatic lectin responsible for selective binding and internalization of galactose/N-acetylgalactosamine-terminating glycoproteins by hepatic parenchymal cells [15]. ASGR plays a role in regulating platelet life span and activation [16], which suggest a potential link of ASGR with coronary disease and acute coronary syndromes. The aquaporin (AQP) family members are fundamental in transmembrane water movements [17]. The specific function of AQP9 is to maximize glycerol influx and urea efflux during gluconeogenesis, as this channel is more permeable to glycerol and urea than to water [18]. Using an AQP9-knockout mice model it has been shown that AQP9 is important for hepatic glycerol metabolism and may play a role in glycerol and glucose metabolism in diabetes mellitus [19].

Early growth response-1 (EGR1) plays a role in the pathogenesis of atherosclerotic lesions, intimal thickening after acute vascular injury, ischemic pathology, angiogenesis, allograft rejection, and cardiac hypertrophy. Aditionally, EGR-1 regulates expression of molecules critically linked with atherogenesis and lesion progression [20], [21], [22]. Peroxisome proliferator-activated receptor G (PPARG), a member of the PPAR subfamily of nuclear hormone receptors, has a key role in adipogenesis, insulin sensitivity, and glucose and lipid metabolism, and also plays a major role in vascular biology, modulating atherosclerosis progression and vascular endothelial function [23], [24]. Killer cell lectin-like receptor subfamily C, member 2 (KLRC2) and member 4 (KLRC4) are expressed primarily in natural killer (NK) cells and are a family of transmembrane proteins characterized by a type II membrane orientation and the presence of a C-type lectin domain. NK cells are an important component of the innate immune system through target cell killing and cytokine production. A direct evidence for NK cell involvement in atherogenesis is scant, although some researchers have localized NK cells to the human atherosclerotic plaque [25], [26]. RNASE2– RNase A family, 2 (liver, eosinophil-derived neurotoxin) is a distinct cationic protein of the eosinophil’s large specific granule known primarily for its ability to induce ataxia, paralysis, and central nervous system cellular degeneration [27]. FCGR1A – Fc fragment of IgG, high affinity 1A, receptor (CD64) is unique within the FCGR family which mediates important immune defense functions by inducing cell surface changes on human leukocytes [28], [29]. Activating FCGRs can induce phagocytosis, antigen presentation, the production of reactive oxygen species (ROS) and cytokines [28].

### Canonical Analysis

Activation of PPAR changes gene transcription to modulate several clinically important metabolic functions: it improves the lipid profile and corrects hyperglycaemia and insulin resistance [30]. Interestingly, PPAR and retinoid × receptor (RXR) cooperate in metabolic regulation. Numerous PPAR agonists are used in clinical setting (fibrates) or are currently under investigation for therapeutic application (thiazolidinediones).

IL-6 seems to have proatherogenic effects. Its serum concentration is elevated in acute myocardial infarction [31] and predicts events in the follow-up [32]. IL-6 has been shown to enhance fatty lesion development in mice [33]. IL-6 signaling is regulated by a family of endogenous JAK kinase inhibitor proteins, suppressors of cytokine signaling (SOCS) [34]. SOCS3 has a differential effect on IL-6 and IL-10 receptor signaling: the IL-6 receptor signaling is down-regulated by SOCS3, whereas that by IL-10 receptor is not [35]. In mice, leukocyte-derived IL-10 prevents the development of advanced atherosclerosis and plays a critical role in the modulation of the cellular and collagen plaque composition [36]. On the other hand, an elevated baseline plasma level of IL-10 has been reported as a strong and independent predictor of long-term adverse cardiovascular outcome in patients with acute coronary syndrome [37].

LXR/RXR plays important roles in cholesterol metabolism and regulates a number of immune and inflammatory pathways that have the potential to modulate development of atherosclerotic lesion [38].

### Study Limitations

Since no data on gene expression in STEMI has been published so far (means, standard deviations, etc.), we conducted a pilot study with a rather small number of patients. The results should be confirmed on a larger and independent cohort. Our group was homogenous (STEMI patients only, with angioplasty of infarct-related artery) and well-characterized clinically. We compared expression with two controls: the same group after six months (this approach has several strengths: age, sex and risk factors are the same, and the STEMI phase can be compared with the subsequent stable phase of the coronary artery disease) and an independent control group with coronary artery disease, but without myocardial infarction. Our aim was to find genes showing changed expression specifically during MI, but not those associated with stable coronary artery disease (that is why we did not take healthy controls). Thanks to such choice also the treatment of both groups was comparable, with the exception of a clopidogrel and – obviously – heparin. Our control group was older than the experimental group, but when we compared age 1^st^ or 4^th^ quartile of the study group with the controls, the major results were the same (data not shown). Obviously the left ventricle ejection fraction was different – but this cannot be avoided. Other clinical factors were not significantly different.

### Conclusions

ST-segment elevation myocardial infarction alters expression of several groups of genes. On admission, several genes and pathways that could be directly or indirectly linked with lipid/glucose metabolism, platelet function and atherosclerotic plaque stability were affected (signaling of PPAR, IL-10, IL-6). Analysis at discharge highlighted specific immune response (upregulation of immunoglobulins). Highly significant and substantial upregulation of SOCS3 and FAM20 genes expression in the first 4–6 days of myocardial infarction in all patients is the most robust observation of our work.

## Supporting Information

Table S1 **Primers sequences and reaction conditions used for Real-time PCR.**
(DOC)

Table S2 **Annotated genes with expression at admission significantly different from 6 months after MI.**
(DOC)

Table S3 **Annotated genes with expression at admission significantly different from control.**
(DOC)

Table S4 **Annotated genes with expression at discharge significantly different from 6 months after MI.**
(DOC)

Table S5 **Annotated genes with expression at discharge significantly different from control.**
(DOC)
