# Supplementary material for: Altered Gene Expression Pattern in Peripheral Blood Mononuclear Cells in Patients with Acute Myocardial Infarction
Source: PLoS One. 2012 Nov 21;7(11):e50054. doi: 10.1371/journal.pone.0050054 (PMC3503717; doi:10.1371/journal.pone.0050054)
Supplement: Table S1 — Primers sequences and reaction conditions used for Real-time PCR. (DOC) [file pone.0050054.s001.doc]

Table S1. Primers sequences and reaction conditions used for Real-time PCR.

| **Gene Symbol** | **NCBI accession for mRNA record** | **Primer sequence 5’→3’** | **Product size (bp)** | **Annealing (ºC)** | **Extension (s)** | **Melting (ºC)** |
| --- | --- | --- | --- | --- | --- | --- |
| ASGR2 | NM_080912 | F: CAGGCACTCGCAGGCTGAAT  R: TGACCACCAGCAGCAGGATG | 144 | 57 | 5 | 89 |
| AQP9 | NM_020980 | F: AGTGAGGACCACAACAGGTA  R: AGCCACATCCAAGGACAATC | 186 | 50 | 5 | 86 |
| CR1 | NM_000573 | F: GTGCCAGGCGGATGACAGAT  R: CGCTGCTGCCTCCTTGAGAA | 215 | 60 | 9 | 79 |
| CYP1B1 | NM_000104 | F: AACGTACCGGCCACTATCAC  R: AAGGAAGGCCAGGACATAGG | 201 | 56 | 9 | 88 |
| DYSF | NM_001130978 | F: GTCTGTCCGCCAGCTTCAAT  R: CATCCAGGTCAGGCAGAGTC | 162 | 56 | 7 | 90 |
| EGR1 | NM_001964 | F: GTGCTGGTGGAGACCAGTTA  R: TCACTAGGCCACTGACCAAG | 145 | 56 | 6 | 90 |
| EGR2 | NM_000399 | F: CACCAGCTGTCTGACAACAT  R: AGATCCAACGACCTCTTCTC | 164 | 56 | 7 | 80 |
| FAM20A | NM_017565 | F: GCGGCTCCTCAATGTCATCG  R: CCTCTGGCGTTGTCAAGGTG | 129 | 60 | 5 | 85 |
| FCGR1A | NM_000566 | F: GGCAGGAACACATCCTCTGA  R: TGGTAACTGGAGGCCAAGCA | 147 | 58 | 6 | 87 |
| FMN1 | NM_001103184 | F: CCAAGAGGATGAGCTGGTTA  R: AGCTCGCGTGATGATCTCTA | 208 | 56 | 9 | 80 |
| HBEGF | NM_001945 | F: ACTGCTACCTCTGAGAAGAC  R: CCAGATACCATCGGACATAC | 176 | 60 | 7 | 83 |
| HP | NM_005143 | F: CCGAGATTGCACATGGCTAT  R: ATCTCCTTCTGTGCGCAGTT | 80 | 60 | 7 | 82 |
| HPRT | NM_000194 | F: TGACCTTGATTTATTTTGCATACC  R: CGAGCAAGACGTTCAGTCCT | 102 | 55 | 5 | 80 |
| KLRC2 | NM_002260 | F: AGTGGTTGCCAATGTCTCAG  R: CGCAGAGTTACAACCATCAC | 121 | 58 | 5 | 82 |
| KLRC4 | NM_013431 | F: TACTACTCAGCCTCCAACAC  R: GCCAGACTCACTTCTGAGTA | 166 | 58 | 8 | 84 |
| MYBL1 | NM_001080416 | F: ATAGCCTTGACGAGCACACT  R: AACGTCACTCCATGCTACAG | 191 | 56 | 8 | 86 |
| PPARG | NM_138712 | F: ATGCTGGCCTCCTTGATGAA  R: TGCCAAGTCGCTGTCATCTA | 171 | 58 | 7 | 85 |
| RNASE1 | NM_198232 | F: GTCCGGCTCCTTCTGCTTGT  R: GTGTCATATTCCGGCGCCTC | 172 | 58 | 9 | 87 |
| RNASE2 | NM_002934 | F: TCCACAGTATCCGGTGGTTC  R: CACAAGGCACTTCAGCTCAG | 195 | 58 | 8 | 85 |
| SOCS3 | NM_003955 | F: AGGCTCCTGGTAGAGAAGAC  R: CCATCCAGGCTGAGTATGTG | 135 | 50 | 5 | 86 |
| STAB1 | NM_015136 | F: GTGCCTGCCTTCAGCCTCTT  R: GTGTCTGCGTCCAGGTGACT | 146 | 60 | 7 | 88 |
| ST14 | NM_021978 | F: CCAACAACCAGCATGTGAAG  R: TGTCGGTGTAGGACTGATCT | 195 | 56 | 8 | 89 |
| TCN2 | NM_000355 | F: GCTCGCTCTCAGAGCCAACT  R: CCGCTTCTGGTGGAGACACA | 184 | 58 | 9 | 86 |
| TMEM176A | NM_018487 | F: CTGGAGCTGCTGCCTTCATT  R: CAGTCACTCGAGCTGGAGAT | 181 | 56 | 8 | 86 |
| TUBB | NM_178014 | F: CTTCAAGCGCATCTCGGAGC  R: TGCGGTGGCATCCTGGTACT | 119 | 61 | 5 | 90 |
| VSIG4 | NM_001184830 | F: GAAGTAGCTCTGGCTGTGAT  R:TGTACCAGCCACTTCACCAA | 194 | 56 | 8 | 85 |
