# Supplementary material for: Altered Gene Expression Pattern in Peripheral Blood Mononuclear Cells in Patients with Acute Myocardial Infarction
Source: PLoS One. 2012 Nov 21;7(11):e50054. doi: 10.1371/journal.pone.0050054 (PMC3503717; doi:10.1371/journal.pone.0050054)
Supplement: Table S2 — Annotated genes with expression at admission significantly different from 6 months after MI. (DOC) [file pone.0050054.s002.doc]

Table S2. Annotated genes with expression at admission significantly different from 6 months after MI. D = duplicate

| Fold Change | p-value | ID | Notes | Symbol | Entrez Gene Name |
| --- | --- | --- | --- | --- | --- |
| −1.578 | 2.30E−06 | 7961182 |  | KLRC2 | killer cell lectin-like receptor subfamily C, member 2 |
| −1.563 | 1.51E−07 | 7961166 |  | KLRC4 | killer cell lectin-like receptor subfamily C, member 4 |
| −1.505 | 1.36E−09 | 8151101 |  | MYBL1 | v-myb myeloblastosis viral oncogene homolog (avian)-like 1 |
| 1.503 | 7.96E−11 | 8098041 |  | TMEM144 | transmembrane protein 144 |
| 1.506 | 5.86E−12 | 8039226 |  | LILRA3 | leukocyte immunoglobulin-like receptor, subfamily A (without TM domain), member 3 |
| 1.518 | 5.04E−13 | 8137264 |  | TMEM176A | transmembrane protein 176A |
| 1.524 | 2.09E−07 | 7940237 |  | MS4A4A | membrane-spanning 4-domains, subfamily A, member 4 |
| 1.526 | 9.29E−12 | 7961440 |  | PLBD1 | phospholipase B domain containing 1 |
| 1.538 | 2.07E−04 | 8054722 |  | IL1B | interleukin 1, beta |
| 1.541 | 3.15E−13 | 8025285 |  | C19orf59 | chromosome 19 open reading frame 59 |
| 1.546 | 1.27E−07 | 7933084 | D | NAMPT | nicotinamide phosphoribosyltransferase |
| 1.547 | 2.16E−08 | 8027862 |  | FFAR2 | free fatty acid receptor 2 |
| 1.549 | 7.00E−17 | 8103415 |  | FAM198B | family with sequence similarity 198, member B |
| 1.559 | 1.81E−03 | 8055952 |  | NR4A2 | nuclear receptor subfamily 4, group A, member 2 |
| 1.559 | 1.18E−07 | 8142120 | D | NAMPT | nicotinamide phosphoribosyltransferase |
| 1.562 | 4.53E−04 | 8034694 |  | mir-24 | microRNA 24-1 |
| 1.567 | 3.59E−10 | 8001457 |  | CES1 | carboxylesterase 1 |
| 1.570 | 1.68E−16 | 7945204 |  | ST14 | suppression of tumorigenicity 14 (colon carcinoma) |
| 1.573 | 5.36E−05 | 8114572 |  | HBEGF | heparin-binding EGF-like growth factor |
| 1.574 | 2.15E−05 | 8043981 |  | IL1R2 | interleukin 1 receptor, type II |
| 1.577 | 5.82E−16 | 8012028 |  | ASGR2 | asialoglycoprotein receptor 2 |
| 1.584 | 5.95E−14 | 8080344 |  | STAB1 | stabilin 1 |
| 1.587 | 3.26E−03 | 7933872 |  | EGR2 | early growth response 2 |
| 1.589 | 1.07E−06 | 7987163 |  | FMN1 | formin 1 |
| 1.590 | 3.06E−11 | 8140840 |  | STEAP4 | STEAP family member 4 |
| 1.591 | 3.82E−05 | 7975779 |  | FOS | FBJ murine osteosarcoma viral oncogene homolog |
| 1.596 | 2.43E−15 | 7938390 |  | ADM | adrenomedullin |
| 1.601 | 6.36E−12 | 7909371 |  | CR1 | complement component (3b/4b) receptor 1 (Knops blood group) |
| 1.604 | 2.14E−09 | 8030860 |  | FPR2 | formyl peptide receptor 2 |
| 1.611 | 3.11E−13 | 7977615 |  | RNASE1 | ribonuclease, RNase A family, 1 (pancreatic) |
| 1.613 | 8.92E−09 | 8162276 |  | NFIL3 | nuclear factor, interleukin 3 regulated |
| 1.618 | 2.28E−12 | 8145736 |  | NRG1 | neuregulin 1 |
| 1.629 | 1.88E−12 | 8072360 |  | TCN2 | transcobalamin II |
| 1.635 | 4.25E−13 | 7905571 |  | S100A9 | S100 calcium binding protein A9 |
| 1.638 | 4.61E−11 | 8173287 |  | VSIG4 | V-set and immunoglobulin domain containing 4 |
| 1.644 | 9.77E−15 | 8044391 |  | MERTK | c-mer proto-oncogene tyrosine kinase |
| 1.651 | 2.12E−07 | 8008885 |  | mir-21 | microRNA 21 |
| 1.656 | 2.54E−11 | 7960794 |  | CD163 | CD163 molecule |
| 1.663 | 3.21E−14 | 8022711 |  | DSC2 | desmocollin 2 |
| 1.669 | 6.74E−12 | 8051583 |  | CYP1B1 | cytochrome P450, family 1, subfamily B, polypeptide 1 |
| 1.673 | 3.26E−03 | 8029693 |  | FOSB | FBJ murine osteosarcoma viral oncogene homolog B |
| 1.683 | 3.42E−13 | 7920238 |  | S100A12 | S100 calcium binding protein A12 |
| 1.686 | 4.53E−04 | 7922976 |  | PTGS2 | prostaglandin-endoperoxide synthase 2 (prostaglandin G/H synthase and cyclooxygenase) |
| 1.693 | 6.24E−13 | 8042637 |  | DYSF | dysferlin, limb girdle muscular dystrophy 2B (autosomal recessive) |
| 1.694 | 7.87E−07 | 8095736 | D | AREG/AREGB | amphiregulin |
| 1.703 | 2.90E−14 | 8103951 |  | ACSL1 | acyl-CoA synthetase long-chain family member 1 |
| 1.761 | 1.99E−11 | 7973110 |  | RNASE2 | ribonuclease, RNase A family, 2 (liver, eosinophil-derived neurotoxin) |
| 1.778 | 9.45E−13 | 8077899 |  | PPARG | peroxisome proliferator-activated receptor gamma |
| 1.880 | 4.21E−11 | 7919133 | D | FCGR1A | Fc fragment of IgG, high affinity Ia, receptor (CD64) |
| 1.891 | 2.08E−10 | 8045688 |  | TNFAIP6 | tumor necrosis factor, alpha-induced protein 6 |
| 1.904 | 9.39E−11 | 7905047 | D | FCGR1A | Fc fragment of IgG, high affinity Ia, receptor (CD64) |
| 1.940 | 9.40E−07 | 8095744 | D | AREG/AREGB | amphiregulin |
| 1.945 | 1.01E−10 | 8017867 |  | FAM20A | family with sequence similarity 20, member A |
| 1.958 | 1.71E−15 | 7983910 |  | AQP9 | aquaporin 9 |
| 2.066 | 5.51E−05 | 8108370 |  | EGR1 | early growth response 1 |
| 2.339 | 9.00E−14 | 7997188 |  | HP | haptoglobin |
| 2.929 | 1.60E−20 | 8018864 |  | SOCS3 | suppressor of cytokine signaling 3 |
