# Supplementary material for: Altered Gene Expression Pattern in Peripheral Blood Mononuclear Cells in Patients with Acute Myocardial Infarction
Source: PLoS One. 2012 Nov 21;7(11):e50054. doi: 10.1371/journal.pone.0050054 (PMC3503717; doi:10.1371/journal.pone.0050054)
Supplement: Table S3 — Annotated genes with expression at admission significantly different from control. (DOC) [file pone.0050054.s003.doc]

Table S3. Annotated genes with expression at admission significantly different from control. D = duplicate

| Fold Change | p-value | ID | Notes | Symbol | Entrez Gene Name |
| --- | --- | --- | --- | --- | --- |
| -3.501 | 4.71E-19 | 7914216 |  | SNHG12 | small nucleolar RNA host gene 12 (non-protein coding) |
| -3.155 | 8.66E-23 | 8097056 |  | SNORA24 | small nucleolar RNA, H/ACA box 24 |
| -3.090 | 3.79E-06 | 8081298 |  | GPR128 | G protein-coupled receptor 128 |
| -2.777 | 1.40E-15 | 8130578 |  | SNORA20 | small nucleolar RNA, H/ACA box 20 |
| -2.617 | 3.72E-05 | 8036755 |  | CLC | Charcot-Leyden crystal protein |
| -2.448 | 4.05E-23 | 7951034 | D | TAF1D | TATA box binding protein (TBP)-associated factor, RNA polymerase I, D, 41kDa |
| -2.308 | 1.07E-11 | 8025584 |  | SNORD105 | small nucleolar RNA, C/D box 105 |
| -2.301 | 5.66E-12 | 8113124 |  | ANKRD32 | ankyrin repeat domain 32 |
| -2.293 | 2.24E-16 | 8049299 |  | SCARNA6 | small Cajal body-specific RNA 6 |
| -2.256 | 6.24E-15 | 8030366 | D | RPL13A | ribosomal protein L13a |
| -2.227 | 1.22E-10 | 7902400 | D | RABGGTB | Rab geranylgeranyltransferase, beta subunit |
| -2.213 | 6.33E-05 | 7961182 |  | KLRC2 | killer cell lectin-like receptor subfamily C, member 2 |
| -2.110 | 4.52E-06 | 7940216 |  | MS4A3 | membrane-spanning 4-domains, subfamily A, member 3 (hematopoietic cell-specific) |
| -2.108 | 1.48E-07 | 8059712 |  | SNORD82 | small nucleolar RNA, C/D box 82 |
| -2.102 | 4.49E-09 | 7998722 |  | SNORD60 | small nucleolar RNA, C/D box 60 |
| -2.081 | 3.30E-18 | 8127987 |  | SNORD50A | small nucleolar RNA, C/D box 50A |
| -2.063 | 6.32E-08 | 8175432 |  | SNORD61 | small nucleolar RNA, C/D box 61 |
| -2.057 | 5.83E-09 | 7899480 |  | SNHG3 | small nucleolar RNA host gene 3 (non-protein coding) |
| -2.053 | 8.31E-20 | 7942594 |  | SNORD15B | small nucleolar RNA, C/D box 15B |
| -2.047 | 1.29E-10 | 8034512 |  | SNORD41 | small nucleolar RNA, C/D box 41 |
| -2.044 | 3.75E-18 | 8091778 |  | SCARNA7 | small Cajal body-specific RNA 7 |
| -2.012 | 5.22E-06 | 7967028 |  | RNU4-2 | RNA, U4 small nuclear 2 |
| -2.006 | 2.14E-05 | 7961166 | D | KLRC4 | killer cell lectin-like receptor subfamily C, member 4 |
| -1.994 | 6.60E-12 | 7953383 |  | SCARNA10 | small Cajal body-specific RNA 10 |
| -1.983 | 1.77E-10 | 8010078 |  | SNORD1C | small nucleolar RNA, C/D box 1C |
| -1.982 | 1.44E-04 | 7961175 |  | KLRC3 | killer cell lectin-like receptor subfamily C, member 3 |
| -1.972 | 7.41E-15 | 8026875 |  | SNORA68 | small nucleolar RNA, H/ACA box 68 |
| -1.946 | 1.78E-02 | 8149116 | D | DEFA1 (include others) | defensin, alpha 1 |
| -1.946 | 1.78E-02 | 8149126 | D | DEFA1 (include others) | defensin, alpha 1 |
| -1.946 | 1.78E-02 | 8149137 | D | DEFA1 (include others) | defensin, alpha 1 |
| -1.946 | 2.00E-12 | 8150877 |  | SNORD54 | small nucleolar RNA, C/D box 54 |
| -1.944 | 4.72E-15 | 7956876 |  | LLPH | LLP homolog, long-term synaptic facilitation (Aplysia) |
| -1.824 | 2.81E-12 | 8161024 |  | RMRP | RNA component of mitochondrial RNA processing endoribonuclease |
| -1.819 | 1.11E-12 | 7951036 | D | TAF1D | TATA box binding protein (TBP)-associated factor, RNA polymerase I, D, 41kDa |
| -1.784 | 1.75E-10 | 8049297 |  | SCARNA5 | small Cajal body-specific RNA 5 |
| -1.770 | 2.35E-10 | 7981181 |  | SNHG10 | small nucleolar RNA host gene 10 (non-protein coding) |
| -1.755 | 3.86E-10 | 7938291 |  | SNORA3 | small nucleolar RNA, H/ACA box 3 |
| -1.752 | 5.80E-10 | 7958253 |  | C12orf75 | chromosome 12 open reading frame 75 |
| -1.747 | 6.99E-08 | 7938329 |  | SNORA23 | small nucleolar RNA, H/ACA box 23 |
| -1.744 | 6.49E-09 | 8156026 |  | CEP78 | centrosomal protein 78kDa |
| -1.733 | 1.07E-04 | 7953892 |  | KLRF1 | killer cell lectin-like receptor subfamily F, member 1 |
| -1.727 | 3.33E-05 | 7961187 |  | KLRC1 | killer cell lectin-like receptor subfamily C, member 1 |
| -1.726 | 7.94E-09 | 7948904 |  | SNORD28 | small nucleolar RNA, C/D box 28 |
| -1.725 | 1.43E-10 | 8117368 | D | HIST1H4A (includes others) | histone cluster 1, H4a |
| -1.725 | 5.62E-05 | 8009241 |  | SNORD104 | small nucleolar RNA, C/D box 104 |
| -1.718 | 5.48E-05 | 8094533 |  | DTHD1 | death domain containing 1 |
| -1.717 | 5.51E-07 | 7948906 | D | SNHG1 | small nucleolar RNA host gene 1 (non-protein coding) |
| -1.716 | 1.83E-16 | 8023392 |  | SNORA37 | small nucleolar RNA, H/ACA box 37 |
| -1.701 | 7.70E-07 | 7908161 |  | C1orf21 | chromosome 1 open reading frame 21 |
| -1.696 | 6.60E-09 | 8076463 |  | RRP7B | ribosomal RNA processing 7 homolog B (S. cerevisiae) |
| -1.694 | 4.33E-06 | 8114938 |  | JAKMIP2 | janus kinase and microtubule interacting protein 2 |
| -1.694 | 2.56E-07 | 8023259 |  | SNORD58A | small nucleolar RNA, C/D box 58A |
| -1.693 | 6.32E-08 | 7984257 |  | RNU5A-1 | RNA, U5A small nuclear 1 |
| -1.689 | 1.23E-10 | 7901048 |  | SNORD46 | small nucleolar RNA, C/D box 46 |
| -1.685 | 1.42E-04 | 8020684 |  | HRH4 | histamine receptor H4 |
| -1.684 | 3.11E-06 | 8120783 |  | MYO6 | myosin VI |
| -1.676 | 2.31E-07 | 7922402 | D | GAS5 (includes EG:14455) | growth arrest-specific 5 (non-protein coding) |
| -1.671 | 1.63E-04 | 7981996 |  | SNORD116-24 | small nucleolar RNA, C/D box 116-24 |
| -1.670 | 5.57E-07 | 8168470 |  | COX7B | cytochrome c oxidase subunit VIIb |
| -1.664 | 7.16E-07 | 8151101 |  | MYBL1 | v-myb myeloblastosis viral oncogene homolog (avian)-like 1 |
| -1.659 | 4.24E-10 | 7943160 |  | SCARNA9 | small Cajal body-specific RNA 9 |
| -1.652 | 2.79E-04 | 8105331 |  | GZMK | granzyme K (granzyme 3; tryptase II) |
| -1.649 | 3.92E-07 | 7942957 |  | PRSS23 | protease, serine, 23 |
| -1.648 | 2.60E-02 | 8069505 |  | C21orf15 | chromosome 21 open reading frame 15 |
| -1.643 | 8.00E-09 | 7948894 |  | WDR74 | WD repeat domain 74 |
| -1.635 | 7.69E-07 | 7961151 | D | KLRC4 | killer cell lectin-like receptor subfamily C, member 4 |
| -1.631 | 3.13E-05 | 7961198 |  | KLRAP1 | killer cell lectin-like receptor subfamily A pseudogene 1 |
| -1.619 | 5.23E-11 | 7964246 |  | SNORD59B | small nucleolar RNA, C/D box 59B |
| -1.616 | 6.35E-11 | 8159004 |  | RPL7A | ribosomal protein L7a |
| -1.613 | 3.29E-09 | 8021183 |  | SCARNA17 | small Cajal body-specific RNA 17 |
| -1.610 | 2.48E-04 | 7945262 |  | JAM3 | junctional adhesion molecule 3 |
| -1.604 | 5.02E-06 | 8057517 |  | NCKAP1 | NCK-associated protein 1 |
| -1.601 | 5.28E-05 | 7953835 |  | KLRG1 | killer cell lectin-like receptor subfamily G, member 1 |
| -1.600 | 2.77E-08 | 7922404 | D | GAS5 (includes EG:14455) | growth arrest-specific 5 (non-protein coding) |
| -1.600 | 1.28E-06 | 7952335 |  | SNORD14E | small nucleolar RNA, C/D box 14E |
| -1.599 | 3.43E-08 | 8127989 |  | SNORD50B | small nucleolar RNA, C/D box 50B |
| -1.596 | 4.66E-05 | 7951351 |  | PDGFD | platelet derived growth factor D |
| -1.595 | 1.46E-03 | 8089015 |  | PROS1 | protein S (alpha) |
| -1.592 | 2.70E-06 | 7948900 |  | SNORD30 | small nucleolar RNA, C/D box 30 |
| -1.591 | 2.95E-03 | 7967030 |  | RNU4-1 | RNA, U4 small nuclear 1 |
| -1.590 | 7.12E-05 | 7953949 |  | KLRD1 | killer cell lectin-like receptor subfamily D, member 1 |
| -1.590 | 3.50E-09 | 7951030 |  | SNORD6 | small nucleolar RNA, C/D box 6 |
| -1.589 | 6.65E-06 | 8006602 | D | CCL4 | chemokine (C-C motif) ligand 4 |
| -1.589 | 2.19E-11 | 8114468 |  | SNORD63 | small nucleolar RNA, C/D box 63 |
| -1.588 | 6.87E-07 | 8044035 |  | IL18R1 | interleukin 18 receptor 1 |
| -1.588 | 3.33E-05 | 8093104 |  | TM4SF19 | transmembrane 4 L six family member 19 |
| -1.587 | 7.65E-06 | 7948444 |  | TCN1 | transcobalamin I (vitamin B12 binding protein, R binder family) |
| -1.585 | 6.35E-08 | 7899392 |  | SCARNA1 | small Cajal body-specific RNA 1 |
| -1.575 | 3.97E-07 | 8133106 |  | SNORA22 | small nucleolar RNA, H/ACA box 22 |
| -1.571 | 5.49E-04 | 8044049 |  | IL18RAP | interleukin 18 receptor accessory protein |
| -1.568 | 1.51E-02 | 8112274 |  | ELOVL7 | ELOVL fatty acid elongase 7 |
| -1.566 | 7.32E-07 | 8023757 |  | CD226 | CD226 molecule |
| -1.563 | 1.35E-06 | 8083260 |  | CPA3 | carboxypeptidase A3 (mast cell) |
| -1.562 | 5.68E-06 | 8081799 |  | TIGIT | T cell immunoreceptor with Ig and ITIM domains |
| -1.558 | 8.03E-09 | 7918857 |  | TSPAN2 | tetraspanin 2 |
| -1.554 | 1.44E-05 | 8006621 | D | CCL4 | chemokine (C-C motif) ligand 4 |
| -1.554 | 1.44E-05 | 8019651 | D | CCL4 | chemokine (C-C motif) ligand 4 |
| -1.554 | 3.26E-07 | 7948908 | D | SNHG1 | small nucleolar RNA host gene 1 (non-protein coding) |
| -1.553 | 1.35E-02 | 8096415 |  | MMRN1 | multimerin 1 |
| -1.550 | 1.03E-10 | 7936637 |  | SNORA19 | small nucleolar RNA, H/ACA box 19 |
| -1.546 | 9.44E-09 | 8014248 |  | SLFN13 | schlafen family member 13 |
| -1.544 | 3.59E-13 | 8101228 |  | CNOT6L | CCR4-NOT transcription complex, subunit 6-like |
| -1.540 | 6.53E-06 | 8108627 |  | VTRNA1-1 | vault RNA 1-1 |
| -1.539 | 2.49E-07 | 7951032 |  | SNORA1 | small nucleolar RNA, H/ACA box 1 |
| -1.538 | 2.20E-03 | 8099471 |  | FGFBP2 | fibroblast growth factor binding protein 2 |
| -1.536 | 1.94E-03 | 8113512 |  | EPB41L4A | erythrocyte membrane protein band 4.1 like 4A |
| -1.536 | 5.91E-07 | 8129482 |  | SAMD3 | sterile alpha motif domain containing 3 |
| -1.532 | 8.76E-12 | 8085287 |  | BRK1 | BRICK1, SCAR/WAVE actin-nucleating complex subunit |
| -1.531 | 9.86E-08 | 8091120 |  | GK5 | glycerol kinase 5 (putative) |
| -1.531 | 2.32E-08 | 8044961 |  | RNU4ATAC | RNA, U4atac small nuclear (U12-dependent splicing) |
| -1.526 | 1.80E-07 | 8030360 | D | RPL13A | ribosomal protein L13a |
| -1.521 | 8.88E-10 | 8120061 |  | ENPP4 | ectonucleotide pyrophosphatase/phosphodiesterase 4 (putative) |
| -1.521 | 1.32E-02 | 8102532 |  | PDE5A | phosphodiesterase 5A, cGMP-specific |
| -1.520 | 1.19E-05 | 7950810 |  | SYTL2 | synaptotagmin-like 2 |
| -1.518 | 4.25E-06 | 8112649 |  | FAM169A | family with sequence similarity 169, member A |
| -1.518 | 1.02E-02 | 8100971 |  | PPBP | pro-platelet basic protein (chemokine (C-X-C motif) ligand 7) |
| -1.518 | 3.02E-10 | 8043276 |  | SNORD94 | small nucleolar RNA, C/D box 94 |
| -1.513 | 1.76E-06 | 8130211 |  | SYNE1 | spectrin repeat containing, nuclear envelope 1 |
| -1.513 | 1.94E-04 | 8139100 |  | TARP | TCR gamma alternate reading frame protein |
| -1.511 | 5.61E-06 | 7940226 |  | MS4A2 | membrane-spanning 4-domains, subfamily A, member 2 (Fc fragment of IgE, high affinity I, receptor for; beta polypeptide) |
| -1.509 | 8.50E-06 | 8006608 | D | CCL4 | chemokine (C-C motif) ligand 4 |
| -1.508 | 2.14E-11 | 8121632 |  | KPNA5 | karyopherin alpha 5 (importin alpha 6) |
| -1.507 | 5.96E-04 | 8124448 | D | HIST1H4A (includes others) | histone cluster 1, H4a |
| -1.502 | 1.13E-08 | 7902398 | D | RABGGTB | Rab geranylgeranyltransferase, beta subunit |
| -1.501 | 8.98E-11 | 8099760 |  | ARAP2 | ArfGAP with RhoGAP domain, ankyrin repeat and PH domain 2 |
| -1.501 | 3.85E-06 | 8106393 |  | F2R | coagulation factor II (thrombin) receptor |
| -1.501 | 6.36E-08 | 8052382 |  | FANCL | Fanconi anemia, complementation group L |
| 1.500 | 4.72E-08 | 8172333 |  | CFP | complement factor properdin |
| 1.500 | 3.37E-12 | 8116096 |  | DDX41 | DEAD (Asp-Glu-Ala-Asp) box polypeptide 41 |
| 1.506 | 3.71E-11 | 7914042 |  | MAP3K6 | mitogen-activated protein kinase kinase kinase 6 |
| 1.507 | 3.25E-11 | 8114593 |  | APBB3 | amyloid beta (A4) precursor protein-binding, family B, member 3 |
| 1.509 | 9.58E-10 | 7940582 |  | BEST1 | bestrophin 1 |
| 1.512 | 1.62E-04 | 8173287 |  | VSIG4 | V-set and immunoglobulin domain containing 4 |
| 1.517 | 1.57E-07 | 8161238 |  | RAB1C | RAB1C, member RAS oncogene family pseudogene |
| 1.518 | 4.67E-09 | 7984952 |  | C15orf39 | chromosome 15 open reading frame 39 |
| 1.521 | 4.02E-13 | 8173299 |  | EDA2R | ectodysplasin A2 receptor |
| 1.523 | 3.65E-05 | 8167971 |  | mir-223 | microRNA 223 |
| 1.524 | 3.97E-07 | 8039453 |  | TMEM150B | transmembrane protein 150B |
| 1.526 | 3.68E-11 | 8005785 |  | KSR1 | kinase suppressor of ras 1 |
| 1.527 | 1.35E-09 | 8006214 |  | ADAP2 | ArfGAP with dual PH domains 2 |
| 1.528 | 7.46E-05 | 7898805 |  | C1QB | complement component 1, q subcomponent, B chain |
| 1.528 | 1.01E-09 | 8063386 |  | CEBPB (includes EG:1051) | CCAAT/enhancer binding protein (C/EBP), beta |
| 1.529 | 3.19E-09 | 8110106 |  | HRH2 | histamine receptor H2 |
| 1.533 | 2.03E-04 | 7909371 |  | CR1 | complement component (3b/4b) receptor 1 (Knops blood group) |
| 1.534 | 6.09E-08 | 8030789 | D | SIGLEC7/SIGLEC9 | sialic acid binding Ig-like lectin 7 |
| 1.536 | 1.04E-06 | 8044574 |  | IL1RN | interleukin 1 receptor antagonist |
| 1.536 | 6.04E-08 | 7963774 |  | ZNF385A | zinc finger protein 385A |
| 1.537 | 3.57E-11 | 8116086 |  | DOK3 | docking protein 3 |
| 1.538 | 8.91E-08 | 8012197 |  | PLSCR3 | phospholipid scramblase 3 |
| 1.540 | 7.82E-13 | 8029050 |  | CCDC97 | coiled-coil domain containing 97 |
| 1.541 | 2.44E-09 | 7995976 |  | CPNE2 | copine II |
| 1.546 | 1.92E-06 | 7898799 |  | C1QC | complement component 1, q subcomponent, C chain |
| 1.549 | 8.47E-12 | 7968126 |  | LSP1 (includes EG:16985) | lymphocyte-specific protein 1 |
| 1.552 | 2.87E-10 | 8162759 |  | TBC1D2 | TBC1 domain family, member 2 |
| 1.554 | 7.04E-23 | 8037152 |  | GSK3A | glycogen synthase kinase 3 alpha |
| 1.555 | 1.48E-03 | 7987163 |  | FMN1 (includes EG:296512) | formin 1 |
| 1.556 | 8.64E-08 | 8065353 |  | THBD | thrombomodulin |
| 1.558 | 1.94E-13 | 8010915 |  | FAM101B | family with sequence similarity 101, member B |
| 1.559 | 1.30E-13 | 8013788 |  | FLOT2 | flotillin 2 |
| 1.565 | 7.11E-13 | 7945666 |  | CTSD | cathepsin D |
| 1.565 | 3.29E-16 | 7946228 |  | TPP1 (includes EG:1200) | tripeptidyl peptidase I |
| 1.568 | 2.10E-14 | 8178790 |  | GPSM3 | G-protein signaling modulator 3 |
| 1.569 | 1.26E-08 | 8036503 |  | RASGRP4 | RAS guanyl releasing protein 4 |
| 1.574 | 1.74E-10 | 8178086 | D | HSPA1A/HSPA1B | heat shock 70kDa protein 1A |
| 1.574 | 1.74E-10 | 8179324 | D | HSPA1A/HSPA1B | heat shock 70kDa protein 1A |
| 1.576 | 1.05E-10 | 8118314 | D | HSPA1A/HSPA1B | heat shock 70kDa protein 1A |
| 1.576 | 1.89E-06 | 7963760 |  | NFE2 | nuclear factor (erythroid-derived 2), 45kDa |
| 1.578 | 4.98E-13 | 7947861 |  | SPI1 (includes EG:20375) | spleen focus forming virus (SFFV) proviral integration oncogene spi1 |
| 1.586 | 3.42E-05 | 8051583 |  | CYP1B1 | cytochrome P450, family 1, subfamily B, polypeptide 1 |
| 1.588 | 8.31E-07 | 8005458 | D | LGALS9B | lectin, galactoside-binding, soluble, 9B |
| 1.597 | 4.30E-07 | 8013450 | D | LGALS9B | lectin, galactoside-binding, soluble, 9B |
| 1.597 | 9.71E-08 | 7999217 |  | ROGDI | rogdi homolog (Drosophila) |
| 1.598 | 4.83E-07 | 8122637 |  | SASH1 | SAM and SH3 domain containing 1 |
| 1.600 | 2.09E-04 | 7912937 |  | PADI2 | peptidyl arginine deiminase, type II |
| 1.605 | 2.50E-02 | 7933872 |  | EGR2 | early growth response 2 |
| 1.609 | 2.04E-08 | 7914950 |  | CSF3R | colony stimulating factor 3 receptor (granulocyte) |
| 1.620 | 7.19E-06 | 7937508 |  | CD151 | CD151 molecule (Raph blood group) |
| 1.620 | 8.40E-04 | 7905060 | D | FCGR1A | Fc fragment of IgG, high affinity Ia, receptor (CD64) |
| 1.626 | 3.67E-07 | 8031238 |  | LILRB4 | leukocyte immunoglobulin-like receptor, subfamily B (with TM and ITIM domains), member 4 |
| 1.627 | 3.11E-08 | 8030782 | D | SIGLEC7/SIGLEC9 | sialic acid binding Ig-like lectin 7 |
| 1.629 | 5.86E-11 | 7941583 |  | RAB1B | RAB1B, member RAS oncogene family |
| 1.631 | 1.25E-03 | 7905047 | D | FCGR1A | Fc fragment of IgG, high affinity Ia, receptor (CD64) |
| 1.641 | 5.12E-09 | 8130993 |  | FAM20C | family with sequence similarity 20, member C |
| 1.655 | 4.64E-04 | 7919133 | D | FCGR1A | Fc fragment of IgG, high affinity Ia, receptor (CD64) |
| 1.661 | 2.82E-12 | 8029465 |  | BCL3 | B-cell CLL/lymphoma 3 |
| 1.667 | 6.16E-06 | 8112476 | D | LOC441081 | POM121 membrane glycoprotein (rat) pseudogene |
| 1.674 | 2.68E-05 | 7977615 |  | RNASE1 | ribonuclease, RNase A family, 1 (pancreatic) |
| 1.678 | 1.30E-26 | 7960689 |  | MLF2 | myeloid leukemia factor 2 |
| 1.684 | 9.86E-06 | 8105989 | D | LOC441081 | POM121 membrane glycoprotein (rat) pseudogene |
| 1.688 | 5.70E-15 | 7972055 |  | KCTD12 | potassium channel tetramerisation domain containing 12 |
| 1.708 | 1.09E-14 | 8131179 |  | TTYH3 | tweety homolog 3 (Drosophila) |
| 1.711 | 1.84E-07 | 8077899 |  | PPARG | peroxisome proliferator-activated receptor gamma |
| 1.726 | 7.90E-07 | 8048432 |  | CYP27A1 | cytochrome P450, family 27, subfamily A, polypeptide 1 |
| 1.726 | 7.02E-06 | 8105935 | D | LOC441081 | POM121 membrane glycoprotein (rat) pseudogene |
| 1.732 | 4.98E-05 | 7983910 |  | AQP9 | aquaporin 9 |
| 1.732 | 1.74E-04 | 8114572 |  | HBEGF | heparin-binding EGF-like growth factor |
| 1.734 | 6.07E-10 | 8028652 |  | ZFP36 | zinc finger protein 36, C3H type, homolog (mouse) |
| 1.740 | 2.15E-13 | 8167334 |  | WAS | Wiskott-Aldrich syndrome (eczema-thrombocytopenia) |
| 1.747 | 8.66E-11 | 8166179 |  | CA5BP1 | carbonic anhydrase VB pseudogene 1 |
| 1.753 | 2.51E-08 | 7945204 |  | ST14 | suppression of tumorigenicity 14 (colon carcinoma) |
| 1.765 | 9.63E-08 | 8005809 |  | LGALS9 | lectin, galactoside-binding, soluble, 9 |
| 1.769 | 1.33E-04 | 7973110 |  | RNASE2 | ribonuclease, RNase A family, 2 (liver, eosinophil-derived neurotoxin) |
| 1.771 | 3.90E-05 | 8042637 |  | DYSF | dysferlin, limb girdle muscular dystrophy 2B (autosomal recessive) |
| 1.837 | 1.40E-06 | 8072360 |  | TCN2 | transcobalamin II |
| 1.915 | 9.75E-03 | 7903765 |  | GSTM1 | glutathione S-transferase mu 1 |
| 1.950 | 1.62E-09 | 8012028 |  | ASGR2 | asialoglycoprotein receptor 2 |
| 1.955 | 4.17E-03 | 8108370 |  | EGR1 | early growth response 1 |
| 1.972 | 2.16E-05 | 8017867 |  | FAM20A | family with sequence similarity 20, member A |
| 1.989 | 3.39E-10 | 8080344 |  | STAB1 | stabilin 1 |
| 2.008 | 4.14E-04 | 7997188 |  | HP | haptoglobin |
| 2.236 | 1.07E-02 | 8137264 |  | TMEM176A | transmembrane protein 176A |
| 2.443 | 5.10E-09 | 8018864 |  | SOCS3 | suppressor of cytokine signaling 3 |
