# Supplementary material for: Altered Gene Expression Pattern in Peripheral Blood Mononuclear Cells in Patients with Acute Myocardial Infarction
Source: PLoS One. 2012 Nov 21;7(11):e50054. doi: 10.1371/journal.pone.0050054 (PMC3503717; doi:10.1371/journal.pone.0050054)
Supplement: Table S4 — Annotated genes with expression at discharge significantly different from 6 months after MI. (DOC) [file pone.0050054.s004.doc]

| Fold Change | p-value | ID | Notes | Symbol | Entrez Gene Name |
| --- | --- | --- | --- | --- | --- |
| 1.510 | 6.18E−05 | 8126269 |  | TREML1 | triggering receptor expressed on myeloid cells-like 1 |
| 1.513 | 6.61E−06 | 8018864 |  | SOCS3 | suppressor of cytokine signaling 3 |
| 1.522 | 1.21E−07 | 8043465 | D | IGKC | immunoglobulin kappa constant |
| 1.542 | 2.77E−08 | 7981724 |  | IGHD | immunoglobulin heavy constant delta |
| 1.550 | 9.51E−09 | 8043459 | D | IGKC | immunoglobulin kappa constant |
| 1.551 | 1.06E−07 | 8043468 |  | LOC652493 | ig kappa chain V-I region HK102-like |
| 1.563 | 4.48E−08 | 7995263 | D | IGHA1 | immunoglobulin heavy constant alpha 1 |
| 1.567 | 2.83E−08 | 8001104 | D | IGHG1 | immunoglobulin heavy constant gamma 1 (G1m marker) |
| 1.576 | 1.74E−08 | 7981740 | D | IGHA1 | immunoglobulin heavy constant alpha 1 |
| 1.577 | 2.31E−06 | 8017867 |  | FAM20A | family with sequence similarity 20, member A |
| 1.598 | 1.40E−07 | 8043360 | D | IGK@ | immunoglobulin kappa locus |
| 1.682 | 5.02E−09 | 8053690 | D | IGKC | immunoglobulin kappa constant |
| 1.695 | 2.66E−08 | 8043431 | D | IGKC | immunoglobulin kappa constant |
| 1.709 | 4.22E−08 | 8043449 | D | IGK@ | immunoglobulin kappa locus |
| 1.714 | 6.51E−09 | 8043438 | D | IGKC | immunoglobulin kappa constant |
| 1.749 | 6.88E−09 | 8043433 | D | IGKC | immunoglobulin kappa constant |
| 1.819 | 1.04E−05 | 8043436 | D | IGKC | immunoglobulin kappa constant |
| 1.914 | 6.44E−08 | 8100827 |  | IGJ | immunoglobulin J polypeptide, linker protein for immunoglobulin alpha and mu polypeptides |

Table S4. Annotated genes with expression at discharge significantly different from 6 months after MI. D = duplicate
