# Supplementary material for: Altered Gene Expression Pattern in Peripheral Blood Mononuclear Cells in Patients with Acute Myocardial Infarction
Source: PLoS One. 2012 Nov 21;7(11):e50054. doi: 10.1371/journal.pone.0050054 (PMC3503717; doi:10.1371/journal.pone.0050054)
Supplement: Table S5 — Annotated genes with expression at discharge significantly different from control. (DOC) [file pone.0050054.s005.doc]

Table S5. Annotated genes with expression at discharge significantly different from control. D = duplicate

| Fold Change | p-value | ID | Notes | Symbol | Entrez Gene Name |
| --- | --- | --- | --- | --- | --- |
| -3.118 | 3.24E-06 | 8081298 |  | GPR128 | G protein-coupled receptor 128 |
| -3.084 | 2.87E-22 | 8097056 |  | SNORA24 | small nucleolar RNA, H/ACA box 24 |
| -3.030 | 2.49E-16 | 7914216 |  | SNHG12 | small nucleolar RNA host gene 12 (non-protein coding) |
| -2.479 | 2.52E-13 | 8130578 |  | SNORA20 | small nucleolar RNA, H/ACA box 20 |
| -2.134 | 5.82E-19 | 7951034 | D | TAF1D | TATA box binding protein (TBP)-associated factor, RNA polymerase I, D, 41kDa |
| -2.095 | 3.85E-13 | 8030366 |  | RPL13A | ribosomal protein L13a |
| -2.086 | 8.97E-10 | 8025584 |  | SNORD105 | small nucleolar RNA, C/D box 105 |
| -2.065 | 7.27E-10 | 8113124 |  | ANKRD32 | ankyrin repeat domain 32 |
| -2.062 | 3.14E-09 | 7902400 |  | RABGGTB | Rab geranylgeranyltransferase, beta subunit |
| -2.034 | 1.67E-19 | 7942594 |  | SNORD15B | small nucleolar RNA, C/D box 15B |
| -2.026 | 3.11E-13 | 8049299 |  | SCARNA6 | small Cajal body-specific RNA 6 |
| -2.005 | 7.56E-07 | 8059712 |  | SNORD82 | small nucleolar RNA, C/D box 82 |
| -1.966 | 3.53E-08 | 7899480 |  | SNHG3 | small nucleolar RNA host gene 3 (non-protein coding) |
| -1.964 | 2.12E-16 | 8127987 |  | SNORD50A | small nucleolar RNA, C/D box 50A |
| -1.960 | 6.93E-08 | 7998722 |  | SNORD60 | small nucleolar RNA, C/D box 60 |
| -1.949 | 1.30E-09 | 8034512 |  | SNORD41 | small nucleolar RNA, C/D box 41 |
| -1.938 | 7.42E-04 | 7961182 |  | KLRC2 | killer cell lectin-like receptor subfamily C, member 2 |
| -1.934 | 1.55E-05 | 7967028 |  | RNU4-2 | RNA, U4 small nuclear 2 |
| -1.915 | 1.33E-14 | 7956876 |  | LLPH | LLP homolog, long-term synaptic facilitation (Aplysia) |
| -1.906 | 7.73E-11 | 7953383 |  | SCARNA10 | small Cajal body-specific RNA 10 |
| -1.904 | 1.04E-06 | 8175432 |  | SNORD61 | small nucleolar RNA, C/D box 61 |
| -1.863 | 3.49E-15 | 8091778 |  | SCARNA7 | small Cajal body-specific RNA 7 |
| -1.848 | 5.48E-13 | 8026875 |  | SNORA68 | small nucleolar RNA, H/ACA box 68 |
| -1.833 | 8.01E-09 | 8010078 |  | SNORD1C | small nucleolar RNA, C/D box 1C |
| -1.833 | 6.50E-11 | 8150877 |  | SNORD54 | small nucleolar RNA, C/D box 54 |
| -1.739 | 1.44E-02 | 8036755 |  | CLC | Charcot-Leyden crystal protein |
| -1.721 | 1.16E-10 | 8161024 |  | RMRP | RNA component of mitochondrial RNA processing endoribonuclease |
| -1.713 | 1.80E-07 | 8168470 |  | COX7B | cytochrome c oxidase subunit VIIb |
| -1.709 | 2.52E-03 | 7961175 |  | KLRC3 | killer cell lectin-like receptor subfamily C, member 3 |
| -1.708 | 8.59E-04 | 7961166 |  | KLRC4 | killer cell lectin-like receptor subfamily C, member 4 |
| -1.705 | 2.07E-09 | 7981181 |  | SNHG10 | small nucleolar RNA host gene 10 (non-protein coding) |
| -1.681 | 2.33E-15 | 8085287 |  | BRK1 | BRICK1, SCAR/WAVE actin-nucleating complex subunit |
| -1.677 | 1.01E-07 | 7984257 |  | RNU5A-1 | RNA, U5A small nuclear 1 |
| -1.674 | 1.67E-15 | 8023392 |  | SNORA37 | small nucleolar RNA, H/ACA box 37 |
| -1.672 | 7.20E-09 | 8049297 |  | SCARNA5 | small Cajal body-specific RNA 5 |
| -1.656 | 4.98E-09 | 7948894 |  | WDR74 | WD repeat domain 74 |
| -1.650 | 1.25E-07 | 8066275 |  | LOC100127886 | uncharacterized LOC100127886 |
| -1.647 | 2.52E-09 | 8117368 |  | HIST1H4A (includes others) | histone cluster 1, H4a |
| -1.642 | 6.21E-03 | 8095744 |  | AREG/AREGB | amphiregulin |
| -1.635 | 1.27E-06 | 8023259 |  | SNORD58A | small nucleolar RNA, C/D box 58A |
| -1.633 | 2.76E-11 | 7964246 |  | SNORD59B | small nucleolar RNA, C/D box 59B |
| -1.629 | 3.11E-12 | 8114468 |  | SNORD63 | small nucleolar RNA, C/D box 63 |
| -1.624 | 4.74E-10 | 8044961 |  | RNU4ATAC | RNA, U4atac small nuclear (U12-dependent splicing) |
| -1.615 | 2.36E-03 | 7940216 |  | MS4A3 | membrane-spanning 4-domains, subfamily A, member 3 (hematopoietic cell-specific) |
| -1.613 | 2.46E-06 | 7938329 |  | SNORA23 | small nucleolar RNA, H/ACA box 23 |
| -1.602 | 6.87E-08 | 7938291 |  | SNORA3 | small nucleolar RNA, H/ACA box 3 |
| -1.599 | 1.15E-06 | 8010082 |  | SNORD1A | small nucleolar RNA, C/D box 1A |
| -1.593 | 1.19E-11 | 7936637 |  | SNORA19 | small nucleolar RNA, H/ACA box 19 |
| -1.585 | 7.26E-09 | 7901048 |  | SNORD46 | small nucleolar RNA, C/D box 46 |
| -1.584 | 6.67E-08 | 7899392 |  | SCARNA1 | small Cajal body-specific RNA 1 |
| -1.575 | 1.25E-08 | 7951036 | D | TAF1D | TATA box binding protein (TBP)-associated factor, RNA polymerase I, D, 41kDa |
| -1.557 | 2.15E-07 | 8096733 |  | SGMS2 | sphingomyelin synthase 2 |
| -1.556 | 7.26E-06 | 7948900 |  | SNORD30 | small nucleolar RNA, C/D box 30 |
| -1.549 | 2.44E-07 | 8168416 |  | USMG5/USMG5P1 | up-regulated during skeletal muscle growth 5 homolog (mouse) |
| -1.546 | 1.55E-09 | 8159004 |  | RPL7A | ribosomal protein L7a |
| -1.544 | 3.82E-05 | 7948906 |  | SNHG1 | small nucleolar RNA host gene 1 (non-protein coding) |
| -1.526 | 4.58E-08 | 7951030 |  | SNORD6 | small nucleolar RNA, C/D box 6 |
| -1.525 | 4.88E-07 | 8127989 |  | SNORD50B | small nucleolar RNA, C/D box 50B |
| -1.524 | 1.42E-07 | 8091922 |  | WDR49 | WD repeat domain 49 |
| -1.521 | 1.47E-03 | 8094533 |  | DTHD1 | death domain containing 1 |
| -1.520 | 2.37E-06 | 8133106 |  | SNORA22 | small nucleolar RNA, H/ACA box 22 |
| -1.517 | 6.28E-06 | 8156026 |  | CEP78 | centrosomal protein 78kDa |
| -1.517 | 2.05E-06 | 8049532 |  | LRRFIP1 | leucine rich repeat (in FLII) interacting protein 1 |
| -1.517 | 1.03E-08 | 7925182 |  | SNORA14B | small nucleolar RNA, H/ACA box 14B |
| -1.514 | 2.26E-02 | 8055952 |  | NR4A2 | nuclear receptor subfamily 4, group A, member 2 |
| -1.514 | 1.74E-05 | 7942957 |  | PRSS23 | protease, serine, 23 |
| -1.510 | 7.79E-11 | 8063903 |  | RPS21 | ribosomal protein S21 |
| -1.509 | 6.63E-05 | 8059708 |  | SNORA75 | small nucleolar RNA, H/ACA box 75 |
| -1.507 | 6.80E-06 | 7948904 |  | SNORD28 | small nucleolar RNA, C/D box 28 |
| -1.506 | 2.07E-03 | 8009241 |  | SNORD104 | small nucleolar RNA, C/D box 104 |
| -1.505 | 1.54E-03 | 7961187 |  | KLRC1 | killer cell lectin-like receptor subfamily C, member 1 |
| -1.505 | 1.91E-05 | 7952335 |  | SNORD14E | small nucleolar RNA, C/D box 14E |
| 1.517 | 3.89E-07 | 8166179 |  | CA5BP1 | carbonic anhydrase VB pseudogene 1 |
| 1.517 | 1.54E-07 | 8161238 |  | RAB1C | RAB1C, member RAS oncogene family pseudogene |
| 1.517 | 2.01E-10 | 8131179 |  | TTYH3 | tweety homolog 3 (Drosophila) |
| 1.530 | 1.85E-02 | 8115327 |  | SPARC | secreted protein, acidic, cysteine-rich (osteonectin) |
| 1.542 | 3.58E-05 | 8012028 |  | ASGR2 | asialoglycoprotein receptor 2 |
| 1.544 | 9.13E-07 | 8148572 |  | LY6E | lymphocyte antigen 6 complex, locus E |
| 1.545 | 6.59E-22 | 7960689 |  | MLF2 | myeloid leukemia factor 2 |
| 1.546 | 2.99E-06 | 8005458 | D | LGALS9B | lectin, galactoside-binding, soluble, 9B |
| 1.552 | 5.55E-14 | 8178790 |  | GPSM3 | G-protein signaling modulator 3 |
| 1.552 | 4.31E-03 | 8064716 |  | SIGLEC1 | sialic acid binding Ig-like lectin 1, sialoadhesin |
| 1.554 | 4.03E-05 | 7898805 |  | C1QB | complement component 1, q subcomponent, B chain |
| 1.567 | 9.90E-07 | 7898799 |  | C1QC | complement component 1, q subcomponent, C chain |
| 1.567 | 2.59E-05 | 7937508 |  | CD151 | CD151 molecule (Raph blood group) |
| 1.568 | 3.32E-05 | 8048432 |  | CYP27A1 | cytochrome P450, family 27, subfamily A, polypeptide 1 |
| 1.572 | 9.32E-07 | 8013450 | D | LGALS9B | lectin, galactoside-binding, soluble, 9B |
| 1.584 | 2.24E-10 | 8167334 |  | WAS | Wiskott-Aldrich syndrome (eczema-thrombocytopenia) |
| 1.586 | 4.11E-10 | 7941583 |  | RAB1B | RAB1B, member RAS oncogene family |
| 1.588 | 3.00E-03 | 8017867 |  | FAM20A | family with sequence similarity 20, member A |
| 1.610 | 7.10E-04 | 8118249 | D | C6orf25 | chromosome 6 open reading frame 25 |
| 1.610 | 4.92E-04 | 7981740 |  | IGHA1 | immunoglobulin heavy constant alpha 1 |
| 1.612 | 3.94E-04 | 7981724 |  | IGHD | immunoglobulin heavy constant delta |
| 1.634 | 2.64E-06 | 8005809 |  | LGALS9 | lectin, galactoside-binding, soluble, 9 |
| 1.654 | 1.44E-04 | 8179313 | D | C6orf25 | chromosome 6 open reading frame 25 |
| 1.667 | 9.46E-04 | 7981732 |  | IGHG1 | immunoglobulin heavy constant gamma 1 (G1m marker) |
| 1.705 | 2.38E-04 | 8178074 | D | C6orf25 | chromosome 6 open reading frame 25 |
| 1.875 | 1.23E-02 | 7903765 |  | GSTM1 | glutathione S-transferase mu 1 |
